# The direct effect of drinking to cope on alcohol problems is not mediated by alcohol consumption: Invariance across gender and countries

**DOI:** 10.1016/j.abrep.2022.100469

**Published:** 2022-11-01

**Authors:** Ruichong Shuai, Adrian J. Bravo, Justin J. Anker, Matt G. Kushner, Lee Hogarth

**Affiliations:** a School of Psychology, University of Exeter, Exeter, United Kingdom; b Department of Psychiatry, University of Minnesota, Minneapolis, MN, USA; c Department of Psychological Sciences, William & Mary, USA

**Keywords:** Drinking to cope, Negative affect, Unique risk, Alcohol harm paradox, Invariance across gender and countries

## Abstract

**Background:**

Drinking to cope with negative affect confers a direct risk of alcohol problems independently of greater alcohol consumption (i.e., confers susceptibility to the alcohol harm paradox). However, it remains unclear whether this risk is common across gender and countries.

**Methods:**

The current study applied path analysis to two cross-sectional samples of 18–25-year-old undergraduate hazardous drinking students recruited from the UK (Study 1; N = 873) and internationally (Study 2; N = 4064 recruited in Argentina, Canada, South Africa, Spain, Uruguay, USA, and England). The Drinking Motives Questionnaire (DMQ) measured drinking to cope with negative affect and drinking to enhance positive affect (i.e., enhancement motives). The Alcohol Use Disorders Identification Test (AUDIT) measured alcohol consumption and problems.

**Results:**

In both studies, drinking to cope with negative affect had a direct effect on alcohol problems (S1: *β* = 0.259, SE = 0.031, *p* <.001; S2: *β* = 0.255, SE = 0.017, *p* <.001), and only a negligible proportion of this effect was mediated by alcohol consumption (S1: 2.58 %, *p* =.550; S2: 0.79 %, *p=*.538). By contrast, drinking to enhance positive affect had a smaller direct effect on alcohol problems (S1: *β* = 0.000, SE = 0.033, *p* =.989; S2: *β* = 0.044, SE = 0.017, *p* =.009), and a substantial proportion of this effect was mediated by greater alcohol consumption (S1: 99.76 %, *p* <.001; S2: 60.36 %, *p* <.001). Crucially, in both studies, the direct effect of drinking to cope on alcohol problems was invariant across gender and countries.

**Conclusions:**

These findings suggest that individuals who endorse drinking to cope with negative affect are uniquely susceptible to the alcohol harm paradox, that is, greater alcohol problems which cannot be explained by greater alcohol consumption, and this susceptibility is common across gender and countries.

## Highlights

• Drinking to cope with negative affect confers a direct risk of alcohol problems.
• This unique susceptibility cannot be explained by consumption level.
• This unique susceptibility is invariant across gender and countries.

## 1 Introduction

Frequent alcohol consumption in early adolescence is a strong prospective predictor of alcohol problems (negative consequences and dependence) in later life (Heron et al., 2012, Percy and Iwaniec, 2007). However, alcohol consumption does not explain all the variance in alcohol problems (Prince et al., 2018), suggesting that additional individual difference variables confer unique risk of developing alcohol problems independently of consumption – the so-called alcohol harm paradox (Boyd et al., 2022, Shuai et al., 2022). Motivational theories of addiction have generated evidence that drinking to cope with negative affect is uniquely associated with alcohol problems but not with greater alcohol consumption, whereas conversely, self-reported drinking to enhance positive experience is uniquely associated with greater alcohol consumption but not with alcohol problems (Anderson et al., 2013, Cooper, 1994, Cooper et al., 1995, Cox and Klinger, 1988, Kassel et al., 2000, Kuntsche et al., 2005, Merrill and Read, 2010, Molnar et al., 2010, Read et al., 2003, Simons et al., 2005, Watkins et al., 2015; for a meta-analysis of 28 association studies see Cooper et al., 2016). These dissociable associations suggest that drinking to cope uniquely contributes to the alcohol harm paradox, i.e., excessive alcohol problems above that predicted by level of consumption, whereas drinking for enhancement only contributes to alcohol problems indirectly via greater consumption. Understanding these dissociable risk pathways may help develop effective screening and intervention strategies.

Several studies using structural equation path analysis have tested the dissociation between drinking to cope versus enhancement in their direct/indirect effects on alcohol consumption and problems. In the original demonstration, Cooper et al. (1995) examined data from 1006 adolescent past-six-month drinkers and 960 adult past-year drinkers. The analysed models were complex, involving additional measures of expectancies and depression symptoms. Nevertheless, drinking to cope showed a direct association with alcohol problems independently of consumption, whereas drinking for enhancement showed only an indirect association with alcohol problems through consumption. Comparably, Merrill et al. (2014) tested longitudinal data collected from 552 college students across two years. Drinking to cope had a direct prospective effect on alcohol problems which was not mediated by alcohol consumption, whereas conversely, drinking for enhancement only had an indirect prospective effect on alcohol problems via greater consumption. Finally, Bresin and Mekawi (2021) conducted systematic, meta-analytic structural equation modeling with k = 254 studies and found in cross-sectional studies that coping motives had a relatively stronger direct effect on alcohol problems and a relatively weaker indirect effect on problems via alcohol consumption, compared to enhancement motives. In contrast, enhancement motives had no statistically significant direct effect on alcohol problems and a relatively stronger indirect effect on problems via alcohol consumption, compared to coping motives.

Although these dissociable direct and indirect pathways between drinking motives and drinking outcomes are well-established, it remains unclear about the replicability and generality of these pathways between gender and countries. In Cooper et al.’s (1995) study, these pathways were stronger for males than females; however, Merrill et al. (2014) found these pathways were invariant across gender. Finally, Bresin and Mekawi (2021) did not test invariance across gender due to the small number of studies reporting correlations split by gender. As far as we are aware, only one cross-cultural study has tested these issues and found that the dissociable pathways were invariant across gender and countries (the U.S., Argentina and Spain - Mezquita et al., 2018). However, this study used the Young Adult Alcohol Consequences Questionnaire (YAACQ, Read et al., 2003) that is specific to alcohol-related problems among college students (e.g., ‘I have gotten into trouble at work or school because of drinking’), which consists of more mild items than dependence-focused measures (such as Alcohol Use Disorder Identification Test (AUDIT), validated by Babor et al., 2001). Given the uncertainty about whether these risk pathways differ between gender and countries, the current study aimed to test pathways into alcohol use disorder symptoms measured with the AUDIT and examine their invariance between gender and countries.

The current study aimed to confirm these dissociable pathways conferred by drinking to cope versus enhancement and test their invariance across gender and countries, to determine their replicability and generality. Two cross-sectional samples of 18–25-year-old undergraduate students who reported hazardous drinking were tested, with Study 1 examining a UK sample (N = 873) and Study 2 examining an international sample (N = 4064 recruited in Argentina, Canada, South Africa, Spain, Uruguay, USA, and England). Drinking to cope and enhancement motives were measured with Drinking Motives Questionnaire (DMQ: Cooper, 1994, Grant et al., 2007) and alcohol consumption and problems were measured with subscales of the Alcohol Use Disorders Identification Test (AUDIT: Babor et al., 2001). Pathways were tested with SEM path analysis using bootstrap standardized error estimation with the drinking to cope and enhancement as predictors, alcohol consumption as the mediator and the alcohol problems as the outcome. It was expected that drinking to cope would have a relatively larger direct effect on alcohol problems, but a smaller direct effect on consumption and a relatively smaller indirect effect on problems via consumption (consistent with the alcohol harm paradox). By contrast, it was expected that drinking for enhancement would have a relatively larger direct effect on alcohol consumption, but a relatively smaller direct effect on alcohol problems and a relatively larger indirect effect on problems via consumption (this pattern of predictions is consistent with Bresin and Mekawi (2021) meta-analysis of cross-sectional studies). Moreover, these dissociable pathways were expected to be invariant across gender and countries, demonstrating their replicability and generality. These findings would suggest that preferential endorsement of drinking to cope relative to enhancement marks unique risk for problematic drinking independently of consumption, revealing drinking to cope as an important susceptibility mechanism contributing to the alcohol harm paradox.

## 2 Study 1

### 2.1 Methods

#### 2.1.1 Participants

Participants were recruited from the Psychology research pool at Exeter and the Facebook page “Overheard at Exeter”. A total of 1023 participants completed the survey, from which 873 were selected based on being aged 18–25 and reporting past year hazardous drinking (defined by AUDIT total score of ≥ 3, which is the minimum criterion for hazardous drinking psychometrically evaluated by Nadkarni et al., 2019). The analytical sample had a mean age of 20.52 (SD = 1.61) and were 64 % female. Mean questionnaire scores are shown in Table 1. Participants provided informed consent, were debriefed and reimbursed with course credits or a £3 Amazon voucher depending on their wishes. All studies were approved by the School of Psychology Research Ethics Committee.

**Table 1:** Sample means (and distribution), Bivariate Pearson correlations matrix and Cronbach’s alpha reliability statics (in brackets) for questionnaire measures in Study 1.

Methods	1	2	3	4	Mean (SD, range)
1 Coping motives	(0.93)				3.46 (2.08, 0–9.34)
2 Enhancement motives	**0.56**	(0.82)			5.61 (2.03, 0.50–10)
3 Alcohol consumption	**0.15**	**0.30**	(0.69)		6.69 (2.27, 1–12)
4 Alcohol problems	**0.34**	**0.31**	**0.57**	(0.76)	7.66 (5.12, 0–23)

*Note.* Significant correlations are emboldened and were determined by a 99 % bias-corrected unstandardized bootstrapped confidence interval (based on 10,000 bootstrapped samples) that does not contain zero. Additionally, *p* values were all < 0.001.

#### 2.1.2 Questionnaires

Drinking Motives were measured with the modified Drinking Motives Questionnaire Revised (DMQR validated by Grant et al., 2007), which contains 28 items describing reasons which might motivate participants to drink, which they endorse on a scale ranging from 0 “never” to 10 “always”. The DMQR contains the following five subscales: drinking to cope with anxiety (e.g. “to relax”), drinking to cope with depression (e.g. “to numb my pain”), drinking for enhancement (e.g. “to get a high”), for conformity (e.g. “to be liked”), and drinking to be social (e.g. “as a way to celebrate”). The coping with anxiety/depression subscales were averaged to create a single “coping motives” score due to their high correlation (*r* = 0.71, *p* <.001), and previous work supporting their aggregation in cross-sectional research (Bravo & Pearson, 2017). Only the drinking to cope and enhancement subscales were used in the analysis justified by the studies outlined in the introduction.

Alcohol consumption and problems were measured with the 10-item Alcohol Use Disorder Identification Test (AUDIT), assessing past 12-month experience (Babor et al., 2001). Factor analytic studies indicate that the AUDIT has two subscales (Doyle et al., 2007, Maisto et al., 2000). The Consumption subscale is assessed by three items: “How often do you have a drink containing alcohol”, “How many standard drinks do you have on a typical day when you are drinking”, and “How often do you have six or more standard drinks on one occasion”. The Problems subscale is assessed by seven items addressing both dependence (e.g., “How often during the last year have you found that you were not able to stop drinking once you had started”) and negative consequences (e.g. “How often during the last year have you failed to do what was normally expected from you because of drinking”).

#### 2.1.3 Analytical plan

IBM SPSS Statistics version 28 was used for data curation. Multivariate outliers were first excluded based on Mahalanobis distance greater than 15, leaving N = 873 (i.e., two participant exclusions). To achieve 80 % power for detecting small mediated effect in bias-corrected bootstrap tests, required sample size is 462 (see Table 3, Fritz & Mackinnon, 2007). Thus, the current sample size of 873 should be more than adequate to test the null hypothesis. Univariate outliers (>1.5 times the interquartile range) were winsorized to match the nearest non-outlying score (four data points for enhancement motives, one data point for alcohol consumption and problems were corrected). These procedures ensured that correlation and mediation analyses were not unduly influenced by multivariate or univariate outliers. Coping and enhancement motives were simultaneously entered as the predictor variables (X), alcohol consumption was the mediator (M), and alcohol problems was the outcome (Y). Multi-group analyses were conducted to test model invariance across gender groups. All analyses were carried out using M*plus* 8.6 (Muthén & Muthén, 2019). Given that chi-square difference test widely used for examining model invariance is sensitive to sample size (Brown, 2015), decrements in the Comparative Fit Index (ΔCFI ≤ 0.010, Cheung & Rensvold, 2002) across more and less constrained models were also examined as a test of invariance.

### 2.2 Results

**Fig. 1:**
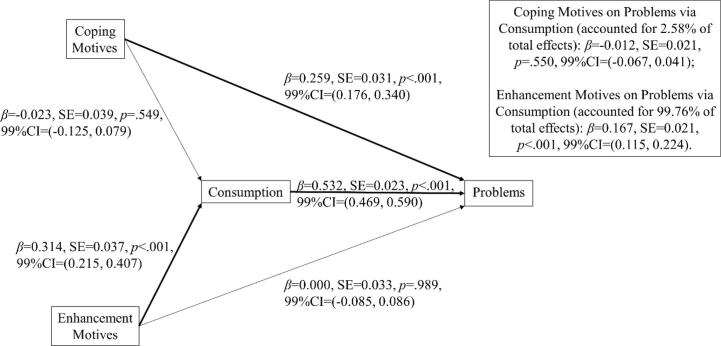
SEM mediation path model in Study 1. Significant parameter estimates are emphasized by emboldened complete connecting lines. As predicted, coping motives had a direct effect on problems and no indirect effect through consumption -> problems pathway, whereas enhancement motives had no direct effect on problems but an indirect effect through consumption -> problems pathway.

Table 1 shows the sample mean, Pearson bivariate correlation matrix and Cronbach’s alpha reliability statistics for the questionnaire measures from Study 1. As expected, all variables correlated with each other. Fig. 1 shows the path model from the SEM analysis revealing the unique direct and indirect associations between variables. As predicted, drinking to cope had a significant unique direct effect on alcohol problems, no significant direct effect on alcohol consumption, and no significant indirect effect on alcohol problems via consumption. By contrast, drinking for enhancement had no significant direct effect on alcohol problems, a significant direct effect on alcohol consumption and a significant indirect effect on alcohol problems via consumption. Furthermore, calculation of the mediation ratio (Preacher & Kelley, 2011) indicated that only 2.58 % of the total effect of coping motives on alcohol problems was explained by the indirect path through consumption. By contrast, 99.76 % of the total effect of enhancement motives on alcohol problems was explained by the indirect path through consumption. Post-hoc power analysis was conducted using Monte Carlo Power Analysis for indirect effects (Schoemann et al., 2017), with inputted correlation coefficients between coping motives, enhancement motives, alcohol consumption and problems (see Table 1). Results showed that the total sample size of 873 achieved 97 % power for detecting indirect effects of coping motives on alcohol problems via consumption and 100 % power for detecting indirect effects of enhancement motives on alcohol problems via consumption, determined by a 99 % bias-corrected unstandardized bootstrapped confidence interval (based on 10,000 bootstrapped samples). Thus, coping motives are unique in having a stronger direct effect on alcohol problems that is not mediated by alcohol consumption, whereas enhancement motives have no unique direct effect on alcohol problems, but increase alcohol problems indirectly via greater alcohol consumption.

Table 2 shows results from the multi-group analysis between gender groups suggesting an adequate fit of the model (MG1). The addition of constraints between the paths of the two gender groups (MG2) indicated that this model was not invariant (ΔCFI = 0.019, greater than the recommended cut-off point 0.01). To identify an invariant model, the path with the greatest contribution to reducing model fit within the fully constrained model was identified and allowed to be freely estimated. The final model obtained (MG3; the path from Enhancement Motives to Consumption) showed ΔCFI = 0.007 compared with the baseline model (MG1), suggesting model invariance between gender groups. In the final multi-group model, the path from Enhancement Motives to Consumption was significant for both gender groups, but stronger for males (*β* = 0.419, SE = 0.051, *p* <.001, 99 %CI = 0.280–0.542) than females (*β* = 0.248, SE = 0.044, *p* <.001, 99 %CI = 0.133–0.360). The rest of paths were invariant between gender groups, specifically, the direct path between drinking to cope and alcohol problems, and the indirect path between drinking for enhancement and alcohol problems via consumption.

**Table 2:** Invariance test results of the SEM across gender.

Mediation Model Across Gender											
		Overall Fit Indices						Comparison Fit Indices			
		χ^2^	df	CFI	TLI	RMSEA	SRMR	Model comparison	Δ*χ^2^*	Δ*df*	ΔCFI
MG1	Unconstrained	0	0	1.000	1.000	0 (0 0)	0	–	–	–	–
MG2	Full constrained model+	15.563**	5	0.981	0.961	0.070 (0.032 0.110)	0.042	MG2 vs MG1	15.563	5	−0.019
MG3	Full constrained model less constraint EC	8.036	4	0.993	0.982	0.048 (0.000 0.096)	0.021	MG3 vs MG1	8.036	4	−0.007

*Note*. **p <.01. + includes the constraints in the paths observed in Fig. 1. The constraints EC refers to the path Enhancement Motives -> Consumption.

## 3 Study 2

### 3.1 Methods

#### 3.1.1 Participants

Participants were recruited from Argentina, Canada, South Africa, Spain, Uruguay, USA, and England, from January 2019 to March 2020. All participants completed a standardized online battery (translated to Spanish for Spanish-speaking participants) of assessments via Qualtrics software. A total of 5674 participants fully completed the survey (see Bravo et al., 2021 for more details). The analytic sample for the present study compromised N = 4069, 18–25-year-old hazardous drinking students who met the same inclusion criteria as in Study 1. The analytic sample had a mean age of 19.53 (SD = 1.65) and compromised 71.9 % female. Table 2 shows the sample mean scores for questionnaires. Participants provided informed consent, were debriefed and reimbursed with course credits. All studies were approved by the School Research Ethics Committees for each institution.

#### 3.1.2 Questionnaires

Participants completed questions assessing age, gender and the AUDIT (the US version: Saunders et al., 1993). AUDIT-US version with the first three items on a 6-point scale was converted to a 5-point scale to match the AUDIT-UK version (see Appendix for details), so that AUDIT scores measuring alcohol consumption and problems were compatible between two studies. Drinking motives were assessed with the four factor Drinking Motives Questionnaire-Revised Short Form (DMQR-SF validated by (Kuntsche & Kuntsche, 2009) consisting of four subscales assessing drinking to cope (e.g. “to cheer up when you are in a bad mood”), to drink for pleasure enhancement (e.g. “to get high”), for conformity (e.g. “to be liked”), and to be social (e.g. “because it improves parties and celebrations”), which participant endorsed on a scale ranging from 1 “almost never/never” to 5 “almost always/always”. DMQR-SF is found to provide nearly equivalent measurement of drinking motives in college student drinkers in comparison to the full-length DMQR (Harbke et al., 2019). As before, only the coping and enhancement scales were analysed. It is important to note that both the AUDIT and DMQR were found to have measurement invariance across all the countries.

#### 3.1.3 Analytical plan

The analytical protocol and predictions were identical to Study 1. Six multivariate outliers were removed by Mahalanobis distance greater than 16.5, leaving N = 4064. As noted in Study 1, the current sample size of 4064 should be more than adequate to test the null hypothesis (N = 462 required for achieving 80 % power for detecting small mediated effect in bias-corrected bootstrap tests, see Table 3 in Fritz & Mackinnon, 2007). Univariate outliers were winsorized to match the nearest non-outlying score (16 data points for DMQR coping, 3 data points for AUDIT consumption and 31 data points for AUDIT problems were corrected). For invariance test across countries, Uruguay and Argentina were grouped into a South America sample (N = 532) as done in previous studies (Pilatti et al., 2021).

**Table 3:** Sample means (and distribution), Bivariate Pearson correlations matrix and Cronbach’s alpha reliability statics (in brackets) for questionnaire measures in Study 2.

Methods	1	2	3	4	Mean (SD, range)
1 Coping motives	(0.81)				1.95 (0.96, 1–5)
2 Enhancement motives	**0.29**	(0.70)			3.04 (1.00, 1–5)
3 Alcohol consumption	**0.10**	**0.35**	(0.63)		5.50 (2.21, 0–11)
4 Alcohol problems	**0.29**	**0.19**	**0.23**	(0.70)	3.60 (3.72, 0–28)

*Note.* Significant correlations are emboldened and were determined by a 99 % bias-corrected unstandardized bootstrapped confidence interval (based on 10,000 bootstrapped samples) that does not contain zero. Additionally, *p* values were all < 0.001.

### 3.2 Results

**Fig. 2:**
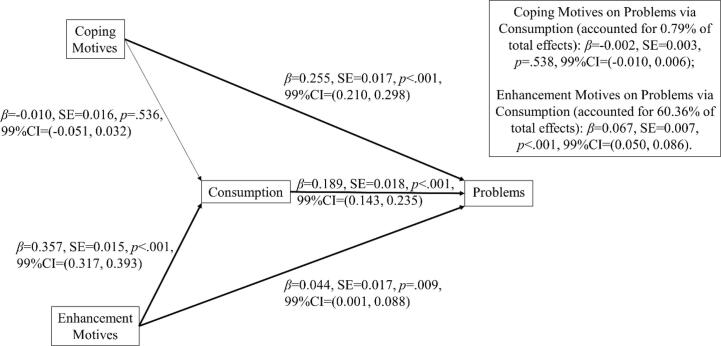
SEM mediation path model in Study 2. Significant parameter estimates are emphasized by emboldened complete connecting lines. Corroborated with Study 1, coping motives had a larger direct effect on problems and no indirect effect through consumption -> problems pathway. Enhancement motives showed a much smaller direct effect on problems compared to coping motives, but similar magnitude to the indirect effect through consumption -> problems pathway.

Table 3 shows sample mean (and distribution), Pearson bivariate correlation matrix, and Cronbach’s alpha reliability statistics for the questionnaires. All variables were correlated as expected. Fig. 2 shows the path model from the SEM analysis revealing the unique direct and indirect associations between variables. Similar to Study 1, drinking to cope had a significant unique direct effect on alcohol problems, no significant direct effect on alcohol consumption, and no significant indirect effect on alcohol problems via consumption. By contrast, drinking for enhancement had a smaller significant direct effect on alcohol problems, a larger significant direct effect on alcohol consumption and a larger significant indirect effect on alcohol problems via consumption. Furthermore, calculation of the mediation ratio indicated that only 0.79 % of the total effect of coping motives on alcohol problems was explained by the indirect path through consumption. By contrast, 60.36 % of the total effect of enhancement motives on alcohol problems was explained by the indirect path through consumption. Post-hoc power analysis was conducted using Monte Carlo Power Analysis for indirect effects (Schoemann et al., 2017), with inputted correlation coefficients between coping motives, enhancement motives, alcohol consumption and problems (see Table 3). Results showed that the total sample size of 4064 achieved 100 % power for detecting indirect effects of coping motives on alcohol problems via consumption and 100 % power for detecting indirect effects of enhancement motives on alcohol problems via consumption, determined by a 99 % bias-corrected unstandardized bootstrapped confidence interval (based on 10,000 bootstrapped samples).

The results from the multi-group analysis between gender groups suggested an adequate fit of the model (MG1A) (Table 4). The addition of constraints between the paths of the two gender groups (MG2A) resulted in minimal change in model fit (ΔCFI = 0.006), suggesting that the dissociable pathways of coping and enhancement motives were invariant across gender. Results from the constrained multi-group model across countries indicated that this model was not invariant across countries (ΔCFI = 0.074, greater than the recommended cut-off point 0.01). To identify an invariant model, the path with the greatest contribution to reducing model fit within the fully constrained model was identified and allowed to be freely estimated (MG3B; the path from Consumption to Problems). Following this, the next path with the greatest contribution to reducing model fit was identified and allowed to be freely estimated (MG4B; the path from Enhancement Motives to Consumption). Finally, the last path was identified (i.e., the path from Coping to Consumption) and the final model obtained (MG5B) showed ΔCFI = 0.004 compared with the baseline model (MG1B), suggesting model invariance across countries. In the final multi-group model, only two paths were constrained, i.e., Coping Motives to Problems and Enhancement Motives to Problems, suggesting that both paths were invariant across countries. Specifically, coping motives showed a greater direct effect on alcohol problems, not via consumption, across all countries. In contrast, the indirect effect of enhancement motives on problems via consumption was only statistically invariant across the USA, Canada, South Africa and England, but not in Spanish-speaking countries (Spain and South America). As reported in Table 5, the path from Enhancement Motives to Consumption was statistically significant across all countries, but the strength was weaker in Spanish-speaking countries compared to the USA, Canada, South Africa and England. The path from Consumption to Problems was statistically significant in the USA, Canada, South Africa and England, but not in Spanish-speaking countries (which explains the non-significant indirect effects for Spanish-speaking countries).

**Table 4:** Invariance testing results of the SEM across gender and countries.

Mediation Model Across Gender											
		Overall Fit Indices						Comparison Fit Indices			
		χ^2^	df	CFI	TLI	RMSEA	SRMR	Model comparison	Δ*χ^2^*	Δ*df*	ΔCFI
MG1A	Unconstrained	0	0	1.000	1.000	0 (0 0)	0	–	–	–	–
MG2A	Constrained	11.763*	5	0.994	0.987	0.026 (0.006 0.045)	0.017	MG2A vs MG1A	11.763	5	−0.006

Mediation Model Across Countries											

		Overall Fit Indices						Comparison Fit Indices			
		χ^2^	df	CFI	TLI	RMSEA	SRMR	Model comparison	Δ*χ^2^*	Δ*df*	ΔCFI

MG1B	Unconstrained	0	0	1.000	1.000	0 (0 0)		–	–	–	–
MG2B	Full constrained model+	101.952***	25	0.926	0.911	0.067 (0.054 0.081)	0.044	MG2B vs MG1B	101.952	25	−0.074
MG3B	Full constrained model less constraint CP	64.993***	20	0.956	0.935	0.058 (0.042 0.074)	0.034	MG3B vs MG1B	64.990	20	−0.044
MG4B	Full constrained model less constraints CP, EC	43.563***	15	0.972	0.945	0.053 (0.035 0.072)	0.025	MG4B vs MG1B	43.563	15	−0.028
MG5B	Full constrained model less constraints CP, EC, CC	13.845	10	0.996	0.989	0.024 (0.000 0.051)	0.014	MG5B vs MG1B	13.845	10	−0.004

*Note*. *p <.05, ***p <.001. + includes the constraints in the paths observed in Fig. 2. The constraints CP refers to the path Consumption -> Problems, EC refers to the path Enhancement Motives -> Consumption, and CC refers to the path Coping Motives -> Consumption.

**Table 5:** Indirect effects of enhancement motives on alcohol problems via consumption in six countries (MG5B).

	*USA*			Canada			South Africa			UK			Spain			South America		
	*β*	SE	99 %CI	*β*	SE	99 %CI	*β*	SE	99 %CI	*β*	SE	99 %CI	*β*	SE	99 %CI	*β*	SE	99 %CI
Enhancement Motives to Consumption	**0.331**	**0.022**	**(0.275,0.388)**	**0.321**	**0.035**	**(0.227,0.409)**	**0.322**	**0.046**	**(0.202,0.436)**	**0.475**	**0.048**	**(0.124,0.453)**	**0.243**	**0.049**	**(0.114,0.367)**	**0.193**	**0.044**	**(0.082,0.307)**
Consumption to Problems	**0.164**	**0.026**	**(0.227,0.318)**	**0.214**	**0.038**	**(0.116,0.308)**	**0.321**	**0.055**	**(0.166,0.453)**	**0.317**	**0.063**	**(0.346,0.590)**	0.098	0.054	(-0.033,0.240)	0.082	0.045	(-0.036,0.193)
Specific indirect effects from Enhancement Motives to Problems via Consumption	**0.054**	**0.009**	**(0.032,0.080)**	**0.069**	**0.015**	**(0.035,0.111)**	**0.103**	**0.024**	**(0.048, 0.175)**	**0.151**	**0.034**	**(0.062,0.236)**	0.024	0.013	(-0.007,0.064)	0.016	0.010	(-0.005,0.047)

*Note*. Significant associations are emboldened and were determined by a 99% bias-corrected unstandardized bootstrapped confidence interval (based on 10,000 bootstrapped samples) that does not contain zero.

### 3.3 Discussion

The current study tested whether the dissociable effects of drinking to cope and enhancement on alcohol consumption and problems were invariant across gender and countries. As predicted, in both studies, drinking to cope had a direct effect on alcohol problems and no indirect effect on problems via consumption, whereas drinking for enhancement had a small direct effect on alcohol problems but an indirect effect via greater alcohol consumption. Calculation of the mediation ratio indicated that the indirect path through consumption accounted for a tiny proportion of the total effect of coping motives on alcohol problems (S1: 2.58 %; S2: 0.79 %), and accounted for a substantial proportion of the total effect of enhancement motives on alcohol problems (S1: 99.76 %; S2: 60.36 %). Importantly, the direct effect of drinking to cope on alcohol problems was invariant across gender and countries, suggesting that the extent to which drinking to cope confers susceptibility to the alcohol harm paradox is comparable across multiple groups. By contrast, the indirect effect of drinking for enhancement on problems via consumption varied between countries, potentially revealing the obscure impact of drinking cultures on the link between consumption and problems (Peele, 1997). Specifically, in Spanish-speaking countries, the link between enhancement motives and alcohol consumption was weaker and the link between alcohol consumption and alcohol problems was not statistically significant. Taken together, these findings suggest that drinking to cope confers a unique risk factor for developing alcohol problems independently of alcohol consumption across gender and countries. In contrast, enhancement motives may lead to alcohol problems via increased consumption across gender. However, this indirect effect appears to be variant across countries, with no statistically significant indirect effect observed in Spanish-speaking countries compared to the other countries.

We found that the direct effect of drinking to cope on alcohol problems was invariant between gender groups. This finding is consistent with studies which have also found this path to be invariant between gender groups (Merrill et al., 2014, Mezquita et al., 2018). However, it contrasts with one study which suggested that this path is stronger in males than females (Cooper et al. 1995), and other studies which have shown that female college students are more susceptible to coping-motivated drinking and its associated problems than male students (Hussong, 2007, LaBrie et al., 2012, Norberg et al., 2010, Rice and Van Arsdale, 2010). Notably, these latter studies had a similar gender ratio (around 60 % females) to the current study, but a smaller sample size (varying between 85 and 354), so the observed discrepancies might be attributed to variance in smaller samples.

The current study also demonstrated that the direct effect of drinking to cope on alcohol problems was invariant across countries, extending the previous cross-cultural study with data collected from a smaller number of countries (Mezquita et al., 2018). By contrast, the indirect effect of enhancement motives on alcohol problems via consumption varied between countries. Specifically, there was a weaker association between enhancement motives and consumption and no statistically significant association between consumption and problems in Spanish-speaking countries. It could be that people in Spanish-speaking countries are less likely to drink for enhancement compared to the other countries, which may lead to weaker associations with consumption rates.

The current cross-sectional paths are consistent with longitudinal studies which have shown that drinking to cope prospectively predicts an increased risk of alcohol problems (Anderson et al., 2013, Crum et al., 2013, Merrill et al., 2014, Watkins et al., 2015). This temporal relationship supports a causal theory in which coping motives contribute to the development of alcohol problems (rather than the reverse direction). The question is why would coping motives confer such risk? Coping motives mediate the link between multiple sources of adversity (including poverty, abuse, trauma, psychiatric symptoms, discrimination etc.) and alcohol use problems (Brenner et al., 2013, Dworkin et al., 2021, Gerrard et al., 2012, Hannan et al., 2015, Mezquita et al., 2014, Topper et al., 2011). Greater cumulative exposure to adversity in disadvantaged groups combined with narrow use of alcohol as a stress-coping strategy could disproportionately exacerbate adversity (perhaps due to the failure to use beneficial problem solving) creating a vicious circle in which alcohol problems grow or are maintained to a higher level over time, even given an equivalent amount of alcohol consumption (Dixon et al., 2009, Guinle and Sinha, 2020, Hogarth, 2022, Kushner and Anker, 2019, McPhee et al., 2020). Alternatively, individuals who have psychiatric comorbidity and drink to cope may have a biological susceptibility, which renders them more sensitive to problematic consequences of drinking, whilst drinking at a comparable level (Anker and Kushner, 2019, Herman, 2012, Menary et al., 2017). To fully understand these risk pathways, more research is needed on the longitudinal antecedents and consequences of drinking to cope, with careful measurement of level of alcohol consumption and problems over time (Kushner et al., 2011, Kushner et al., 2012).

Several limitations are noteworthy. First, cross-sectional data precludes causal inferences or claims about temporal order. Our data only review incremental predictive validity between psychometric constructs, which may or may not reflect longitudinal effects (Rohrer et al., 2022). Second, Bresin and Mekawi (2021) demonstrated that coping motives had an indirect effect on alcohol use via problems, but we did not test this model in order to focus the theoretical narrative on the alcohol harm paradox. This may limit the comprehensiveness of the models tested, but we hope that the narrow focus will promote understanding of the alcohol harm paradox. Third, we used different versions of DMQR in the two studies. Although these are generally considered as equivalent due to the similarity of the items, we know of no studies that have directly compared them. Fourth, we have only reported two categories for gender, which ignores the multiplicity of gender identities. There was insufficient data in the categories of “Transgender”, “Other” and “Prefer not to respond” for meaningful statistics, but it is important to note that gender is not exclusively binary.

In conclusion, the current study suggested there are distinct risk pathways for individuals who endorse drinking to cope versus enhancement. Whereas drinking to cope confers a direct risk of alcohol problems which is not mediated by greater consumption (i.e., susceptibility to the alcohol harm paradox), drinking for enhancement is related to alcohol problems largely via greater alcohol consumption. Invariance analysis indicated that the direct effect of drinking to cope on alcohol problems is comparable across gender and countries, whereas the indirect effect of drinking for enhancement on alcohol problems via consumption is variant across countries (non-significant in Spanish-speaking counties). Longitudinal research is needed to fully understand exactly why drinking to cope marks a direct risk of alcohol problems.

## Funding

This research was supported by an Alcohol Change UK grant [RS17/03], and a Medical Research Council award [MC_PC_MR/R019991/1] to Lee Hogarth.

## CRediT authorship contribution statement

**Ruichong Shuai:** Conceptualization, Methodology, Investigation, Data curation, Formal analysis, Writing – original draft. **Adrian J. Bravo:** Formal analysis, Writing – review & editing. **Justin J. Anker:** Writing – review & editing. **Matt G. Kushner:** Writing – review & editing. **Lee Hogarth:** Conceptualization, Methodology, Data curation, Formal analysis, Writing – review & editing, Supervision.

## Declaration of Competing Interest

The authors declare that they have no known competing financial interests or personal relationships that could have appeared to influence the work reported in this paper.

## Data Availability

Data will be made available on request.
