# Supplementary material for: The direct effect of drinking to cope on alcohol problems is not mediated by alcohol consumption: Invariance across gender and countries
Source: Addict Behav Rep. 2022 Nov 1;16:100469. doi: 10.1016/j.abrep.2022.100469 (PMC9640946; doi:10.1016/j.abrep.2022.100469)
Supplement: Supplementary data 1 [file mmc1.docx]

## Appendix

Conversion for the AUDIT scales between the US and UK version (the first three items).

| 1. How often do you have a drink containing alcohol? | US | 0 | 1 | 2 | 3 | 4 | 5 | 6 |
| --- | --- | --- | --- | --- | --- | --- | --- | --- |
|  |  | Never | Less than Monthly | Monthly | Weekly | 2-3 times a week | 4-6 times a week | Daily |
|  | UK | 0 | 1 | | 2 | 3 | 4 | |
|  |  | Never | Monthly or less | | 2-4 times a month | 2-3 times a week | 4 or more times a week | |
| 2. How many drinks containing alcohol do you have on a typical day when you are drinking? | US | 0 | 1 | 2 | 3 | 4 | 5 | 6 |
|  |  | 1 drink | 2 drinks | 3 drinks | 4 drinks | 5-6 drinks | 7-9 drinks | 10 or more drinks |
|  | UK | 0 | | 1 | | 2 | 3 | 4 |
|  |  | 1 or 2 drinks | | 3 to 4 drinks | | 5 or 6 drinks | 7,8 or 9 drinks | 10 or more drinks |
| 3. How often do you have 5 or more drinks (if male) OR 4 or more drinks (if female) on one occasion? | US | 0 | 1 | 2 | 3 | 4 | 5 | 6 |
|  |  | Never | Less than Monthly | Monthly | Weekly | 2-3 times a week | 4-6 times a week | Daily |
|  | UK | 0 | 1 | 2 | 3 | 4 | | |
|  |  | Never | Less than monthly | Monthly | Weekly | Daily or almost daily | | |
